# Fibre supply for breeding sows and its effects on social behaviour in group-housed sows and performance during lactation

**DOI:** 10.1186/s40813-020-00153-3

**Published:** 2020-06-05

**Authors:** Miriam Priester, Christian Visscher, Michaela Fels, Karl Rohn, Georg Dusel

**Affiliations:** 1 Department of Life Sciences and Engineering, University of Applied Sciences Bingen, Berlinstraße 109, 55411 Bingen, Germany; 2 Institute for Animal Nutrition, University of Veterinary Medicine Hannover, Foundation, Bischofsholer Damm 15, 30173 Hannover, Germany; 3 Institute for Animal Hygiene, Animal Welfare and Farm Animal Behaviour, University of Veterinary Medicine Hannover, Foundation, Bischofsholer Damm 15, 30173 Hannover, Germany; 4 Institute for Biometry, Epidemiology and Information Processing, University of Veterinary Medicine Hannover, Foundation, Bünteweg 2, 30559 Hannover, Germany

**Keywords:** Animal welfare, Digestibility, Faecal consistency, Feeding, Litter size, Reproduction, Satiety, Swine

## Abstract

**Background:**

Fibre sources as feed components with specific physical characteristics like a high swelling capacity (SC), viscosity and water-binding capacity (WBC) have been discussed to affect sow behaviour and to have long-term effects on lactational performance. The present studies aim to analyse the effects of different fibre sources in diets for sows on behaviour in gestation, reproductive performance as well as piglet development.

**Methods:**

Twenty-eight feedingstuffs (four grain varieties, 16 by-products, three oilseeds and five leguminous plants) were compared concerning swelling capacity, viscosity and water binding capacity to select fibre sources with optimal physical characteristics. Following this a digestibility study was carried out with eight castrates for determining digestibilities of gross energy, crude protein, crude fibre, crude fat and crude ash. Additionally, a practical feeding experiment during gestation was performed with 96 sows of Danish genetics. Two supplements for sows with different fibre sources were composed, namely a control diet (based on wheat bran and lignocellulose) and a test diet containing sugar beet pulp, alfalfa, rapeseed meal, soybean hulls, grape pomace and lignocellulose. Six pens with eight sows each were video-monitored for 2 weeks (evaluation of interactions and fights). Furthermore, the animals were subjected to weekly scoring to count skin injuries. To check the fibre effect on reproductive performance and piglet development, the body condition development of the sows as well as the number and weight of live and stillborn piglets, litter weight- and weaning weight of the litters were recorded.

**Results:**

Digestibility of crude fibre increased significantly in the experimental group (58.8% ± 3.3 vs. 49.0% ± 4.3, *p* = 0.01). At the sow trial, there was a tendency to observe less aggressive interactions and fewer fights in sows in the fibre group without significance. No significant differences could be measured between the two groups concerning performance parameters of sows and piglets.

**Conclusion:**

Only changing the fibre source in a gestational diet does not have significant effects on the sows´ behaviour and performance of sows and piglets in lactation. It should be investigated how the amount of fibre can be increased without having any negatives effects on the performance so that the positive effects on the behaviour of the sows become more obvious.

## Background

Keeping sows in groups has been the usual and mandatory type of housing for sows in the EU since 2006 (§30- paragraph 6 Animal Welfare Livestock Husbandry Regulation (German designation: Tierschutz-NutztierHaltungsverordnung)). Keeping sow groups constant has positive effects on the social behaviour of the animals, whereas regrouping the sows regularly has negative effects [1, 2]. During the production cycle, the sows are regrouped after insemination by reassembly, departures and heat in each lactation. This leads to more aggression and restless groups. In general, regrouping and feeding in the group leads to stress in sows [3]. Liquid feeding systems are used in many conventional swine production systems. A previous study [4] shows that when comparing liquid and dry feeding, the sows in the liquid feeding system are less aggressive during the feeding period. In addition, the time sows spent at the trough is the same with or without partitions in liquid feeding systems. Furthermore, the feed must contain at least 8% crude fibre (CF) or a feed intake of at least 200 g CF/sow and day up to 1 week prior to farrowing has to be achieved (§30- paragraph 6 Animal Welfare Livestock Husbandry Regulation). The standard used classification of fibre sources in feedstuffs like CF, seems not to be sufficient to allow any statement to be made about the quality of the raw material or feed in swine diets. The swelling capacity (SC), water binding capacity (WBC) or water holding capacity (WHC) values can be used for fibre characterisation [5]. In general, the digestibility of the fibre varies according to its botanical origin. The highest dietary fibre (DF) digestibility values are obtained with high pectins and/or low lignin and/or high soluble DF levels (i.e. sugar beet pulp) and the lowest with high lignin and high cellulose levels in DF (i.e. straw). However, it is difficult to use the DF composition as a predictor for digestibility in practice, mainly because it ignores physical interactions between fractions or the structure of DF [6]. The different WHC and viscosity of the different CF components influence the duration of the sows’ feed intake as well as the duration of gastric emptying [7, 8]. In addition, a high WHC and SC provide an increased surface area for the microbes to attach to and digest the fibres [6]. The age of the pigs also has an influence. Fattening pigs that are not yet fully grown do not have a fully developed colon [9] or do not possess the same microbiome as adult swines [10], which is necessary for splitting the vegetable fibre components. In adult sows, the digestibility of the fibre is much higher due to the more mature colon and its microbes [6].

Contrary to the above studies, it was shown that liquid feeding in fattening facilities reduces the feed intake time [11]. Animals also expressed more unwanted behaviour in terms of belly nosing and restlessness after feeding [11]. A fibre-rich diet seems to lower this level of aggression [12]. Furthermore, the available energy and nutrient uptake is an essential factor modulating satiation. Thus, adding fibre to sow nutrition is only effective if the energy and nutrient supply are identical to those of a conventional diet [13]. In order to prevent the body condition score (BCS) of sows from getting too high during gestation, sows are fed restrictively during this time. This leads to a high motivation for food [14, 15] and decreased resting time [16]. The postprandial satiation is increased by the consumption of bulky fibre [17, 18]. A stable body condition is important for the fertility and longevity [19]. Pigs that were kept in natural outdoor enclosures could self-determinedly search for food like roots and spent a large part of their daily activity eating. In comparison, those animals living on conventional production farms only spent 5% of their time with feed intake [20, 21]. A feeding system such as bottom or trough feeding can put sows in a chronic stress situation in which they are exposed to stress for longer than a described threshold period of 2 days. When fed ad libitum, sows can be fed up to 13 times within 24 h [22], or spend time feeding as long as they wanted to (on average 1.5 h per day) [23]. Furthermore, sows indicated strong diurnal and bimodal feeding activity rhythms evolving toward two distinct feeding periods occurring from 05:00 to 09:00 and from 14:00 to 18:00 [24]. Nonetheless, restricted feeding regimens are adopted in commercial practice to maintain an almost constant body-condition and limit fat deposition of sows throughout the reproductive cycle. To meet energy requirements, feed allowances vary between 2 and 2.5 kg, which is approximately 50 to 60% of ad libitum intake [15, 25].

Therefore, restrictive feeding appears to be in contrast to the natural form of feeding, which may result in more atypical behaviour with regard to feeding times [24]. The feeling of satiety and reduction in feelings of hunger, which is insufficient by restrictive feeding of the sows, is the adjusting factor where a positive change can be made concerning animal nutrition. Restricting natural behaviours offers room for the development of new, undesirable behaviours. A fibre-rich diet reduces activity and oral stereotypies. Furthermore, it increases chewing time and resting time [16] and lowers the intake rate [12]. This effect increases over several pregnancies [26]. A high fibre diet alleviates the negative effects of hunger due to reduced exploratory behaviour, higher levels of short-chain fatty acids in the blood and lower levels of the digestive hormone ghrelin [27]. The higher faecal consistency indicates a higher intestinal activity [28].

It was shown that piglets have a higher weight gain due to more stable and higher milk yield of the sows fed with a high fibre diet [12, 28]. There are numerous studies which prove that the well-being of sows can be influenced positively by the targeted use of an increased fibre amount in the feed. In group-housed sows, a good way of reducing stereotypies is to increase the fibre content and overall volume of feed and decrease the energy content [3]. Unfortunately, experiments dealing with digestibility of fibre and other nutrients in sows fed high-fibre diets, [29] reported that these can reduce the fibre constituent digestibility and decrease energy and protein utilisation, suggesting that problems with under-nutrition could occur during late gestation in sows.

With this background, the aim of the present study was to design a fibre diet for sows based on the physical parameters SC, viscosity and WBC, which had the same energy content of a common diet. Furthermore, we wanted to investigate whether an improvement in the digestibility of the high quality fibre diet can be verified in a digestibility trial and whether the behaviour and performance of sows can be improved when using the test diet on-farm.

## Methods

**Fig. 1:**
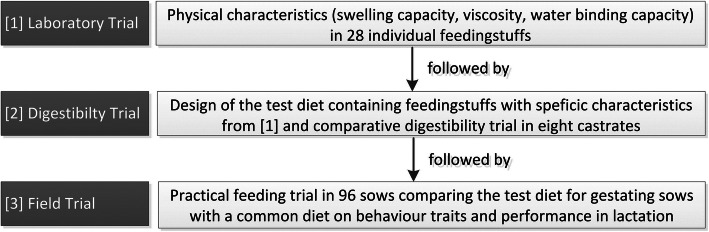
Trial flow diagram. The diagram indicates the sequence of successive tests

The study was carried out within the framework of the EIP-Agri project “Animal welfare – an innovative feeding concept for pigs”, of the German federal state of Rhineland-Palatinate. With this background, the aim of this study was to design a diet for sows with a special fibre concept, focusing on the physical parameters SC, viscosity and WBC (Table 1). The diet was based on the requirements of gestation and lactation diets for sows in accordance with the German norm [30]. For this approach, 28 individual feedstuffs were analysed and described with regard to their physical properties (Laboratory trial, Fig. 1). Based on this analysis, a compound diet for gestating sows was designed and a comparative digestibility trial (control: common diet for pregnant sows) with eight castrates (German Landrasse*Pietrain) was performed (Digestibility trial, Fig. 1). Finally, a practical feeding trial was carried out testing the two diets with regard to the behaviour of the sows and their performance (Field trial, Fig. 1).

**Table 1:** Overview of the feeding mixture

Feed components	CG (%)	FG (%)
Alfalfa	–	15.0
Sugar beet pulp	15.0	21.5
Brewer’s yeast	2.0	–
linseed expeller	2.0	–
Grape pomace	–	7.0
Malt germs	–	3.0
Lignocellulose	–	5.0
Rapeseed meal	–	10.0
Soybean hulls	–	7.0
Soybean meal	25.0	20.1
Vegetable oil	1.0	1.0
Wheat middlings	5.0	–
Wheat bran	42.6	–
Minerals, vitamins and amino acids^a^	7.4	10.4

Vitamin Premix 1% (supplied the following per kg of diet: 8850.00 IU of vitamin A, 900.00 IU of vitamin D3, 672 IU of vitamin D 25-hydroxycholecalciferol, 99 mg of vitamin E, 1.88 mg of vitamin B1, 6.50 mg of vitamin B2, 5.38 mg of vitamin B6, 29.75 mg of vitamin B12, 1.07 mg of vitamin K3, 37.50 mg of niacin_amide acid, 16.25 mg of calcium-D-pantothenic acid, 691.24 mg of choline chloride, 0.04 mg of biotin, 2.70 mg of folic acid, 150 mg of Fe, 13.50 mg of Cu, 1.35 mg of I, 67.50 mg of Mn, 100 mg of Zn, 0.30 mg of Se)

^a^5.0% of calcium carbonate, 1.0% of monocalcium phosphate, 1.0% of sodium chloride, 1% of beet molasses, 0.54% of lysin (HCl), 0.35% of L-threonine, 0.05% of methionine-hydroxy analog

### Laboratory trial

In total, 28 samples of different feeding components, which were available at our compound feed factory, were analysed regarding physical characteristics like SC, viscosity and WBC capacity with established methods. Availability, palatability and cost-effectiveness were taken into consideration. The feeding components were selected without regard to their classification into, for example, fibre or protein sources and included various types of cereals, sugar beet pulp, alfalfa, soy products, rapeseed meal and broad beans (Table 2).

**Table 2:** Values of the WBC, viscosity and SC in %. In addition, the CF values of the feed components are shown. Sugar beet pulp, soybean hulls and grape pomace have overall high values and were selected for the FG on the basis of the measurement results and economic efficiency

Feed components	WBC %	Viscosity mPas	CF %	SC %
**Alfalfa**	589	1.19	27.76	338
**Barley**	323	1.61	4.43	188
**Blue lupin**	462	1.48	14.78	313
**Broad bean**	318	1.62	8.27	256
**Corn**	243	0.98	2.13	125
**Corn germs**	404	1.11	12.37	200
**Corn meal**	288	1.02	3.26	175
**Linseed expeller**^**b**^	607	43.25	9.28	400
**Grape pomace**	422	1.46	16.67	188
**Malt germs**	778	1.07^a^	15.37	313
**Oat fibre**	395	1.10	21.37	175
**Peas**	267	1.57	6.51	213
**ProtiGrain®**	374	1.51	6.77	225
**Rape-seed meal**	319	1.21	12.25	225
**Soybean hulls**	549	1.76	34.31	313
**Soybean meal**	418	1.46	5.15	363
**Soybean meal**	490	1.83	4.76	388
**Soybean meal High Protein**	420	1.35	3.92	250
**Soyabeans**	405	1.24	7.49	163
**Sugar Beet pulp**	989	1.22^a^	12.78	675
**Sunflower meal**	450	1.23	21.81	363
**Triticale**	294	1.69	2.56	163
**Wheat**	258	1.50	2.80	150
**Wheat bran**	504	1.34	10.59	188
**Wheat flour**	331	1.86	4.07	175
**Wheat middlings**	683	1.36	12.10	188
**White lupin**	449	1.54	12.81	275
**Yellow corn gluten**	261	0.98	0.76	200

^a^Sampling at a ratio of 1:10 (dilution), as no result could be determined at a dilution of 1:5

^b^measurement at 5 rpm

Measurement of the SC of the feed was performed in accordance with the method by Robertson and de Monredon [31] with modifications. The method was standardised for each sample, measuring the equivalent to 1 mL of water, in a cylinder filled with 8 mL of distilled water. The cylinder was covered at room temperature for 24 h. The increase in volume of the individual feeds and the remaining water level were noted. The residual water was decanted and weighed back. A floating layer of small particles was observed in some samples. The excess water had to be pipetted off below the floating layer in order to be able to weigh it back. The experiment was repeated and the measurement results of the same feed were arithmetically averaged.

The extract viscosity of feeding stuffs was determined in accordance with the description by Dusel [32]. An amount of 0.2 g of material (ground to sift through a 1.0 mm sieve, ZM 200, Retsch GmbH, Haan, Germany) was put in 10 centrifuge tubes and these were filled with 1 g of distilled water to form a 1:5 suspension. These samples were inoculated in a shaking water bath (30 min, at 38 °C, GFL 1083, LaboTec GmbH & Co.KG, Wiesbaden, Germany) and then centrifuged (3 min, 13.000 rpm, EBA 12, Andreas Hettich GmbH & Co. KG, Tuttlingen, Germany). Within the next 15 min, the extract viscosity was measured by pipetting off and mixing the supernatant from a total of 10 tubes of the same material. Samples of 0.5 mL each were taken and measured at a spindle speed of 100 rpm in the Brookfield Digital Viscometer (MODEL DV-II + VISCOSIMETER, Brookfield, Engineering Laboratories Inc., Stoughton, MA, USA.). For one sample, the spindle speed had to be reduced to 5 rpm due to significantly increased viscosity. The results were then recorded and could be arithmetically averaged. Linseed expeller, malt germs and sugar beet pulp were diluted at a ratio of 1:10 as no result could be determined at a ratio of 1:5.

The WBC of the feed was measured in accordance with the description by De Vries [33]. To measure the WBC, 0.4 g of the material (ground to be sifted through a 1.0 mm sieve) was weighed and filled with 10 g of distilled water. The sample was left covered for 24 h at room temperature and then centrifuged (20 min, 3100 rpm., Hettich Universal 16A Centrifuge, Andreas Hettich GmbH & Co. KG). The supernatant water was decanted in the test tubes and weighed back. A floating layer of small particles was observed even after centrifugation. Here, the excess water had to be pipetted off below the floating layer in order to weigh it back. The following formula was used to calculate the WBC:

$$ \frac{\left(10\mathrm{g}\ \mathrm{of}\ \mathrm{the}\ \mathrm{water}\ \mathrm{quantity}\ \mathrm{used}-\mathrm{decanted}\ \mathrm{water}\ \mathrm{quantity}\ \mathrm{in}\ \mathrm{g}\right)}{\left(0.4\mathrm{g}\ \mathrm{sample}\ \mathrm{weighed}\times 100\right)} $$

All samples were analysed at least in duplicate and results were arithmetically averaged.

### Digestibility trial

The digestibility study was carried out at the test facility of the Technical University in Bingen, Germany. For this purpose, eight castrates (German Landrasse*Pietrain; average take-off body-weight 98 kg) were used - four in each variant.

#### Diets

For the digestibility trial and the subsequent field trial, a complete feed was designed in accordance with the results of the laboratory trial with regard to the physical characteristics of the fibre-rich components. The declaration of the supplement used, which was used on the farm, was reproduced identically in the experimental variant. Feedstuffs, which showed high values in the preliminary tests, were selected. The availability, taste, content of secondary plant substances, energy and protein content also had to be taken into account in the composition. Therefore, one fibre fraction and not a single fibre source was used. This diet was compared to a common diet for pregnant sows used in the field (control diet). The control diet consisted of 55.0% barley, 14.3% wheat, 0.7% vegetable oil and 30.0% of a complementary feed for gestating sows (Schauma EG- TR30, H. Wilhelm Schaumann GmbH, Pinneberg, Germany). In accordance with the declaration, the sow supplement had a CF content of 12.2% and contained mainly wheat bran, and sugar beet pulp as fibre sources (as seen in Table 1). For the complete feed for the experimental group (Fibre group (FG)), the complementary feed (30%) was designed in accordance with the results of the laboratory trial (high SC, viscosity and WBC) by a local company (Mischfutter Werke Mannheim GmbH, Mannheim, Germany). The sow supplement of the experimental variant accordingly contained primarily following crude fibre sources: sugar beet pulp, alfalfa, soybean hulls, grape pomace and lignocellulose (C5, Agromed Austria GmbH, Kremsmünster, Austria; Table 1)**.** The other components were identical to the complete feed in the Control group (CG).

#### Animal housing

The pigs were placed individually in single pens on slatted floors and with pen dividers that allowed for visual contact between pigs. No approval for an animal experiment project in accordance with § 8 Paragraph 1 of the Animal Welfare Act was required for this type of housing according to the National Chemical Investigations Office (Landesuntersuchungsamt- LUA, Koblenz, Department 23). Water was made available to the animals 24 h via nipple drinkers. For each animal, the feed was weighed in variant-individual buckets. All pigs were given an amount of feed so that a complete feed intake was guaranteed; leftover feed was weighed back before the following feeding. In a 4-d long adaptation phase, the pigs were adapted to the experimental diet and the handling by a supervisor. Feed was given in meal form twice daily (1.0 kg at 07:00, 1.0 kg at 16:00).

#### Collection of samples

The bag technique in accordance to van Kleff and Deuring [34] was used to collect quantitative faecal samples from each individual animal. Velcro was glued around the anus of the pigs. As a counterpart, a plastic bag was fastened to the animal with velcro and exchanged after defaecation. The collection period lasted 6 days. All samples were collected, weighed, frozen, pooled and homogenised. Duplicate 150.0 g samples were freeze-dried and ground (to be sifted through a 1.0 mm sieve; centrifugal mill, Retsch ZM200) to determine the dry mass. Samples were analysed for residual dry mass, crude ash, crude protein, crude fat, CF and energy using a Weender analysis as the VDLUFA standard method [35]. The digestibility was calculated with the following formula: [VQ] = (Input-Output)/Input. All analyses were carried out in duplicate.

### Field trial

The practical trial was carried out on a conventional sow farm (Danish genetic) and artificial insemination was carried out with Pietrain.

#### Animal housing

In the gestation unit, the sows stood on slatted flooring with an average space of 2.25 square metres/animal. As occupation material in accordance with §26 paragraph 1 of the Animal Welfare Ordinance, chains with soft wood pendants, as manipulable objects, were placed in the pens. The animals were randomised into two equal groups and placed in 12 pens/ eight sows each (Control group, Fibre group, Fig. 1). In order to prevent air and light influence, the variants were placed in pens facing each other. During the whole gestation period, artificial lighting was provided from 05:00 to 17:00, at the back wall of the stable there were three windows (90x90cm). The average ambient temperature was set at 20 ± 2 °C (mean ± s.d.). A data logger (BL 30, Trotec GmbH, Heinsberg, Germany) was used to record the actual temperature in the gestation barn. The data logger was 35.0 cm above the ground and had to be installed outside the pens on the central passageway. The pollutant gas concentrations of carbon dioxide (CO2), hydrogen sulphide (H2S) and ammonia (NH3) were measured weekly using a pollutant gas measuring device (MultiRAE Lite, RAE Systems Inc., CA, USA) (limit values in accordance with the Animal Welfare Livestock Husbandry Regulation, German designation: TierSchNutztV: NH3–20.0 ppm., CO2–3000.0 ppm., H2S-5.0 ppm). The measurement was carried out in the middle of the respective pen, 35 cm above the ground. From mating until the 5th week of gestation, all sows were fed a standard gestation diet. From the 5th week of gestation until 1 week before farrowing, they were allocated to the two experimental diets. Both diets were provided in meal form once a day at 09:30 in an open trough bymeans of a liquid feeding system (WEDA M16, WEDA Dammann & Westerkamp GmbH, Lutten, Germany).

#### Grouping

Ninety-six sows participated in the trial, bisected into the two variants with six replicates of eight animals depending on the diet (Control group, Fibre group, Fig. 1). The individual pens were sorted according to the age of the sows (previous number of litters), BCS and back fat layer (BFL). This resulted in one group with older sows (4–5 previous lactations), one group of gilts (0 lactations), one young group (1 previous lactation) and three groups of sows with 2–3 previous lactations. The BCS was assessed by means of the visibility and palpability of the spinous processes; hip cusps and pelvic bones were assessed. The fat tissue at the base of the tail and the visibility of fat folds on the inner thigh were included in the classification. A scale of 1–5 (1 = very thin, 2 = thin, 3 = normal, 4 = fat and 5 = very fat) [36] was used to gauge the BCS. The back-fat digital indicator (Renco LEAN-Meater**®,** Renco Inc., Minneapolis, USA) probe was placed on the back of the sows at the level of the last rib, 6.0 cm to the side of the backbone. Three different points at a distance of 15.0 cm were measured and the mean value was taken. The test period included the gestational period (5th week after mating) up until 1 week before lactation (7 days before the expected time of parturition, sows were moved in farrowing crates to a parturition room where they stayed until weaning).

**Fig. 2:**
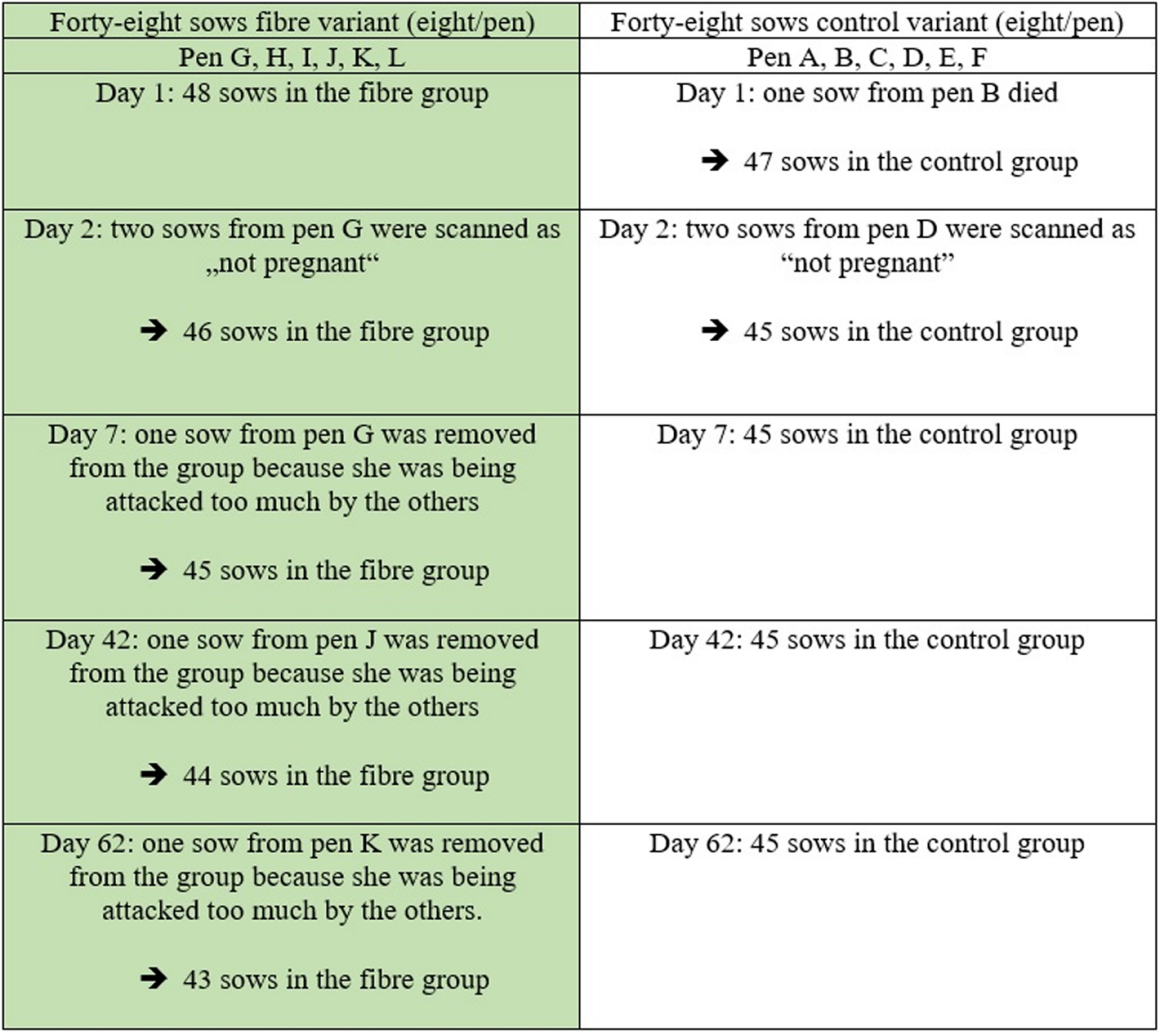
Representation of the group sizes of the sows in the corresponding feed variant. Due to non-pregnancy, death and massive attacks by other sows, animals were separated from the existing groups by day 62 of the experiment

In order to avoid mixing sows again, after rehousing to the gestation stable, some sows had to be excluded from the experiment (Fig. 2). After establishing a rank in the group, no new sow could be placed in the pens. Appropriately, some sows were excluded and 88 experimental sows remained in the experiment of the initial 96 experimental animals up until farrowing.

#### Behaviour observation

**Fig. 3:**
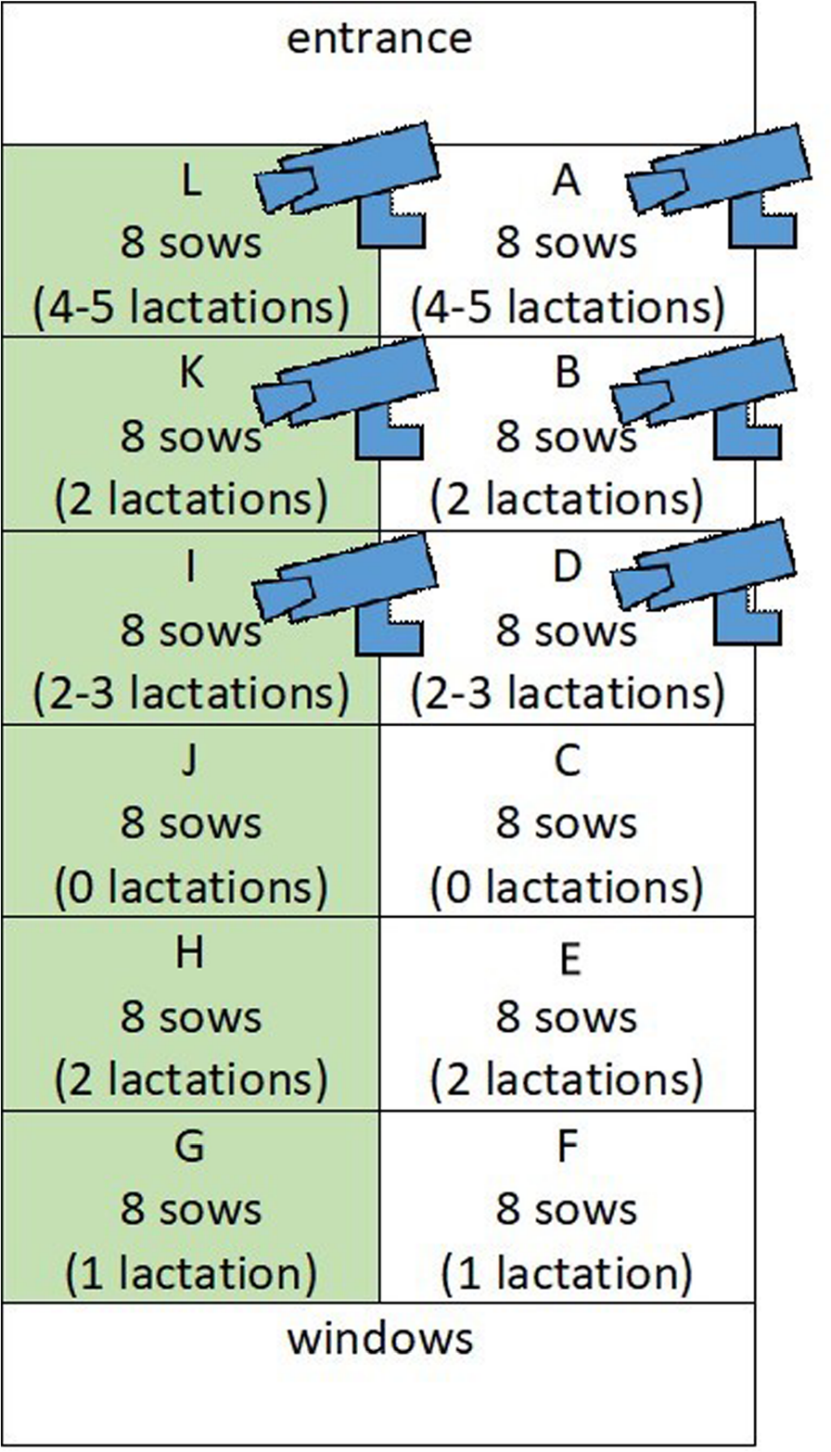
Arrangement of the two feeding groups in the waiting stable (FG = green, CG = white). Division of the pens according to the parity of the sow. Sows of the same age with different feed groups were raised opposite each other. The first three pens were monitored by video

In order to document the social behaviour of the sows, a scoring of skin injuries took place once a week. The right and left sides of the animals from the nose to the tail were examined as one unit. The sows’ snout, legs and vulva were not included in the scoring. The scoring was based on the KTBL guidelines [37]. Injuries with a length of 5.0 cm or a diameter of more than 2.5 cm were counted. Animals with < 5 injuries were given a score of 0, with 5–15 injuries a score of 1 and > 15 injuries a score of 2. In addition, three pens/feed variants were each monitored by video (Mobotix 6MP, MOBOTIX AG, Langmeil, Germany) over a two-week period from 09:00–12:00 with a frequency image recording of 410 images/minute (Fig. 3). Each sow was labelled with an individual number using markers, so that they could be identified on the video recording. The interactions of the animals were then documented.

The pattern of the evaluation of the sows’ behaviour was based on the definition of the agonistic behaviour of the piglets´ ranking order by M Fels, J Hartung and S Hoy [38]. Between two activities, a minimum interval of 30 s was prescribed. Due to the amount of interactions in our study during feeding, this interval was reduced to 20 s. In addition, offensive actions such as biting or head shoving by the sows ended either with escape or with counterattack, resulting in two differently evaluated actions. Therefore, the two following definitions were established:

- Criteria of interaction: the “interaction” as criterion in the video evaluation was characterised by the fact that a sow tried to move another sow away from the spot by biting, head striking or pushing with the help of the snout. To meet this criterion, the second sow was not allowed to react to the first sow’s interaction. The sow that started the “interaction” was recorded in the evaluation as offender, the attacked animal as victim.
- Criteria of fight: the “fight” as criterion in the video evaluation was characterised as one sow’s interaction and a second sow’s reaction. Also here, the animal which had initiated the “fight” was classified as offender and the attacked, defending animal as victim.

#### Faecal consistency

The individual faecal consistency was examined before the start of the trial. During the trial, the faecal consistency was measured from four sows per pen in the waiting stable and finally from all sows in the farrowing barn at weaning. The animals were encouraged to stand up or walk around in the waiting barn to trigger the urge to defaecate. Normally, after the sow had stood up, the defecation took place after a few minutes. Faeces were manually sampled from the rectum if there was no defecation. Sensory assessment of the consistency was carried out using a numerical scale of 1–5 (1 – hard, 5 - liquid) and a consistency measurement with a penetrometer (PNR 6, Anton Paar GmbH, Graz, Austria). The penetrometer measured the penetration depth of a defined test cone with a certain weight into the faeces in accordance with established methods [39]. The penetration depth was described in mm. Two measurements were taken per faecal sample, the values of which were then averaged.

#### Performance data in lactation

With the transfer of gestating sows to the farrowing unit, data collection started on an individual basis. To record the performance parameters of the sows, the animals were monitored at the time of farrowing with a span of 4 days for 24 h by a team of two persons each. To determine the duration of birth, the birth of each individual piglet was recorded. If the birth of a piglet was overlooked and several animals were discovered at once in a pen, + 15 min were calculated per piglet. The birth duration was defined as the time from the birth of the first piglet to the birth of the last piglet. After the placenta had been delivered, the number of piglets born alive, the litter weight, the number and total weight of piglets born dead and the number of mummies were recorded. After the end of the farrowing of all sows, litters were equalised. Cross-fostering was performed for all variants. At the time of weaning, the number of weaned piglets per sow and the weight of the weaned litter were recorded. Individual feed intake of sows was recorded. In addition, scoring of the sows’ shoulders in accordance with the KTBL guidelines [37] was performed on shoulder lesions (0 - no lesion, 1 - reddening/swelling, 2 - fresh open/crusted shoulder lesion).

### Statistical analysis

All statistical analyses in this study were performed using SAS® Enterprise Guide®, Client Version 7.1 (SAS Institute Inc. Cary, NC, USA). The level of significance was set at 0.05 in all models.

If not otherwise specified, all results were expressed as means ± standard deviation (SD). The animals were grouped according to age (previous number of litters), BCS and BFL. The experimental diets were the independent variables.

The skin lesions were scored and analysed using a *Chi*-*Square analysis.*

The data from the behaviour observation including interactions and fights, differentiated into offenders and victims were not normally distributed and were analysed by non-parametric (ANOVA) models, such as Wilcoxon two-sample test, Wilcoxon rank-sum and Kruskal-Wallis tests.

The performance parameters from the farrowing, including length, number and weight of piglets born alive, number and weight of stillborn piglets, number of mummies, number and weight of weaned piglets, were analysed by non-parametric (ANOVA) models with Kruskal-Wallis tests.

## Results

The experiments (Laboratory trial, Digestibility trial, Field trial) ran without complications in accordance with the experimental protocol. In the following, the results are presented in three sections.

### Laboratory trial

The physical characteristics were tested on a total of 28 different feed materials.

#### Swelling capacity

**Fig. 4:**
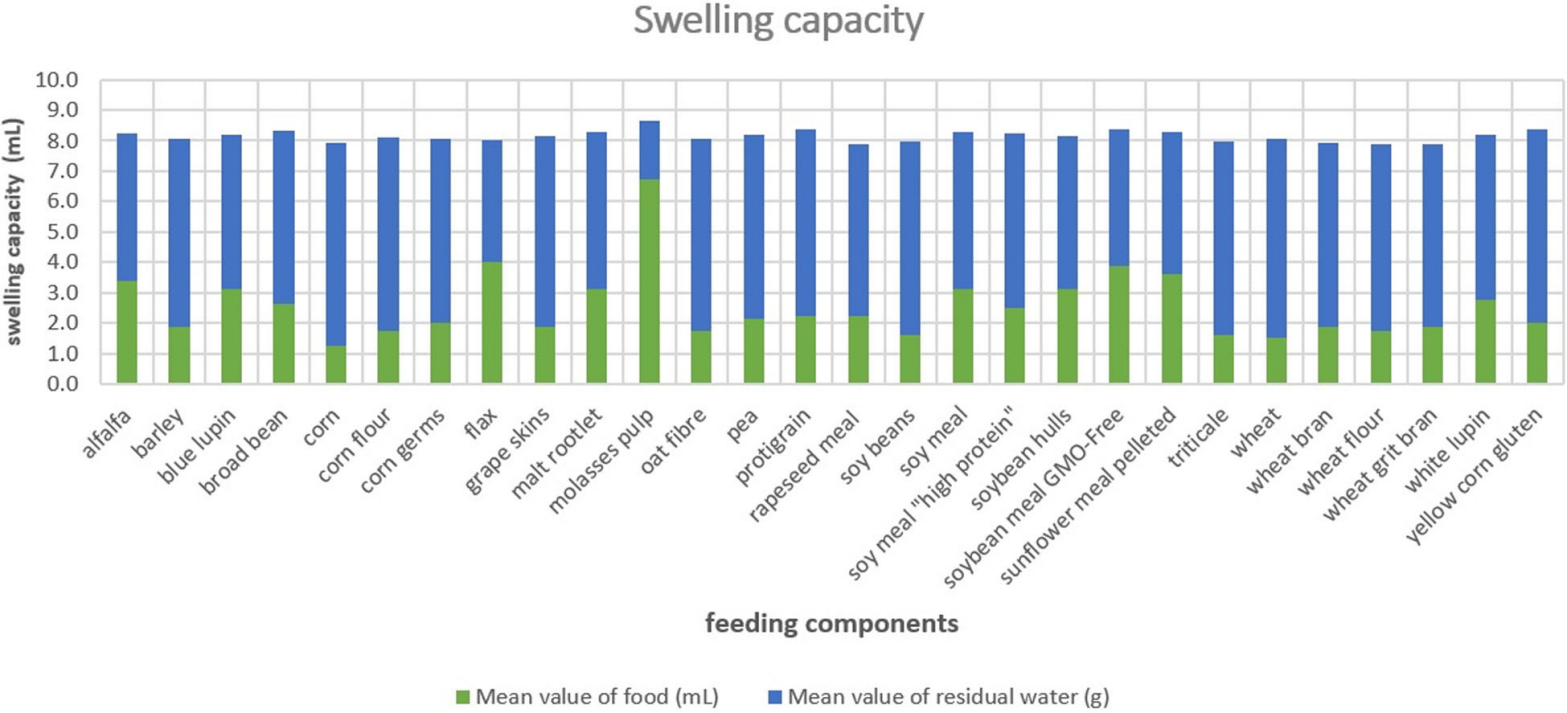
Swelling capacity of various components used in pig feeding. The high swelling capacity of the sugar beet pulp is notable, which is why it was selected as one of the fibre sources in the FG

Of the 28 fibre sources investigated, percentage values of swelling capacity were measured from 125% (corn) to 675% (beet pulp). The two main components of the CG supplement were wheat bran (188% SC), soybean meal (250% SC). Due to the high SC- values, the fibre sources alfalfa (338% SC), soybean hulls (313% SC) and rapeseed meal (225% SC) were selected for the FG. The following Fig. 4 shows the values of food in mL and residual water in g. The maximum value of beet pulp is clearly visible due to its high SC.

### Viscosity

The higher the viscosity, the thicker the fluid. Tables 2 and 3 shows the results of the measurements in a mixing ratio of 1:5. The viscosity values were between 0.98 mPas (corn, yellow corn gluten) and 43.5 mPas (linseed expeller). The fibre source, soybean hulls (1.76 mPas) were above the mean value of the examined components when the outlier value of linseed expeller was ignored. The fibre sources, malt germs (1.07 mPas) and sugar beet pulp (1.22 mPas) were also selected due to their high viscosity. The values appear low, but the samples had to be measured in a dilution of 1:10 because otherwise the values would have been outside the measurable range.

**Table 3:** Analysed feed ingredients of the control and experimental variant. The protein content between the two groups was kept relatively constant. The crude fibre content of the experimental variant was almost 3% higher than in the control variant

Ingredients	CG- feed	FG- feed	Sow supplement CG	Sow supplement FG
**Crude protein %**	13.3	13.3	17.6	20.4
**Crude fiber %**	6.3	9.1	11.2	15.9
**Crude fat %**	1.9	2.7	2.2	2.6
**Crude ash %**	3.7	5.3	12.4	12.4
**Gross energy MJ/kg**	16.7	16.4	15.8	15.9
**Dry matter %**	91.1	88.9	92.2	91.3

### Water-binding capacity (WBC)

The WBC describes the amount of water that the fibre can retain after an external force has been applied. The more water a feed can retain, the higher its swelling capacity. The WBC values were between 243% (corn) and 989% (sugar beet pulp). The values of the main components of CG were relatively high with 504% wheat bran, 418% soybean meal and 683% wheat middlings. As in the other physical parameters, sugar beet pulp had the highest measured value with 989% and therefore made up the largest share with 21% in the supplement in the FG. The values of the malt germs (778%) and alfalfa (589%) were decisive for the use of these individual components. Grape pomace (422%) and soybean hulls (549%) were selected due to the high WDC value in combination with the Weender crude fibre determination values. Even though the focus was on selecting in accordance with physical parameters, CF values of 34.31% (rape seed meal) and 16.67% (grape pomace) were not disregarded.

### Digestibility trial

#### Composition of complete feeds

The composition of both experimental diets was determined in the laboratory at the University of Applied Sciences Bingen, Germany in accordance with the Weender analysis. All measurements were performed twice. The analysis of the sow supplements clearly showed that the values of CF in the FG were higher than in the CG. Also, the values in the FG feed were higher than in the CG feed. The values of the feed mixtures CG/FG feed were composed of the two different supplements (30%), plus the barley and wheat from own cultivation. The same feed mixtures as in the digestibility study were also used in the sow experiment.

The apparent digestibility of gross energy as well as crude protein, crude fat and crude ash showed no significant differences between the two tested feed variants. However, the digestibility of the CF was significantly higher in the group of pigs fed a fibre diet as shown in Table 4.

**Table 4:** Apparent digestibility coefficient of nutrients of nursery pigs fed the two diets for sows with different fibre components

	Gross energy (%)	Crude protein (%)	Crude fiber (%)	Crude fat (%)	Crude ash (%)
CG (Mean)	83.5	83.5	49.0^a^	73.4	69.4
CG (SD)	1.3	1.2	4.3	6.3	9.7
FG (Mean)	81.6	80.5	58.8^b^	73.5	76.2
FG (SD)	1.8	2.7	3.3	4.4	3.6
*P*-value	0.13	0.11	**0.01**	0.98	0.26

### Field trial

The relevant parameters for the description of the external conditions in the barn were documented by various measurements.

#### Housing conditions in gestation

The average temperature in the barn was 23.7 °C. The weekly measured values did not reach, let alone exceed the upper limit in any pen for any harmful gas. All the measured pollutant gas values were below the average permitted upper limit. In §26 (General Requirements for the Keeping of Pigs) of the Animal Welfare Livestock Husbandry Regulation (Tierschutz Nutztierhaltungs VO), stipulated values for harmful gases are listed which must not be exceeded permanently. The following values apply: NH3 -20 ppm/m^3^, CO2 -3000 ppm/m^3^ and H2S - 5 ppm/m^3^. The average values recorded were NH3–7.9 ppm/m^3^ (±1.3). CO2–640.3 ppm/m^3^ (±111.1) and H2S − 0.1 ppm/m^3^ (±0.0).

#### Behavioural observation

The weekly scoring of the skin injuries showed no significant differences between the two feed groups (*p* = 0.11).

When the three recorded hours were considered individually and the differences between the two groups were evaluated, significant differences in the first hour of interactions (offender CG 17.74 vs. FG 30.00, *p* = 0.0021, victim CG 16.07 vs. FG 31.60, *p* = < 0.0001) and in the third hour of interactions (offender CG 28.04 vs. FG 20.13, *p* = 0.0477, victim CG 29.13 vs. FG 19.08, *p* = 0.0120), as well as in the number of victims in the fight were noticeable (victim CG 27.65 vs. FG 20.50, *p* = 0.0424; Table 5).

**Table 5:** Video evaluation data presenting a two-week period. The recordings were made daily between 09:00 and 12:00. Shown are the average scores of the Wilcoxon test and the *p*-values of the Kruskal-Wallis test. Over the entire three-hours feeding period, no significant differences in offender or victim interactions and fights were observed

	Interaction “offender” Mean Score	Interaction “victim” Mean Score	Fight “offender” Mean Score	Fight “victim” Mean Score
CG	25.09	24.26	25.98	25.67
FG	22.96	23.75	22.10	22.40
*Pr > ChiSq*	0.595	0.898	0.318	0.395

#### Faecal consistency

The faecal consistency of the sows was not significantly changed by the use of different fibre components. Between the first measurement at the beginning of the experiment and the second measurement in the middle of the pregnancy, the faecal consistency in both groups became softer (Table 6). At the last week before farrowing, the faecal consistency in both groups became harder again.

**Table 6:** Data of faecal consistency, measured once in the penetrometer PNR6 and tested once sensorially

Date	Consistency penetrometer [mm]*		Consistency sensoric tested (Score 1–5)**	
	CG	FG	CG	FG
Day 29 of gestation	62.3	57.3	2.3	2.5
*p*-value	0.454		0.966	
Day 71 of gestation	80.0	85.0	2.8	2.9
*p*-value	0.699		0.700	
Day 107 of gestation	63.5	62.0	3.0	3.0
*p*-value	0.836		0.801	

*The values are given in mm for the penetration depth (high value equals soft consistency). The harder the faecal consistency, the lower the value measured in PNR6

** The sensory tested (st) evaluation from 1 (hard), 3 (physiologically) to 5 (liquid)

#### Condition of the sows during reproduction cycle

The BCS of the sows hardly varied between the two different feed groups. The sows started with mean scoring values of 3.0 (±0.5) in the CG vs. 3.0 (±0.6) FG 1 week before farrowing and being rehoused from the waiting stable to the farrowing pens (*p* = 0.802). Even after farrowing, there were no significant differences between the two feed groups with mean scoring values of 2.8 (±0.4) CGF vs. 2.9 (±0.3) FG (*p* = 0.545).

The BFL values as well as the BCS values show that there were no significant differences in the body conditions of the sows between the two groups. One week before farrowing, we measured values of 19.8 mm (±5.7) CG vs. 20.0 mm (±4.9) FG (*p* = 0.890). Immediately before weaning, the sows naturally had lower BFL values of 15.9 mm (±4.6) CG vs. 16.7 mm (±4.0) FG (*p* = 0.347).

The number of shoulder lesions after weaning showed no significant differences. From a total of 43 animals in the CG, eight animals received the score 2 (open shoulder lesion) and five animals the score 1 (skin reddening/swelling). The remaining animals showed no signs of shoulder lesion and scored as 0. In the FG, from a total of 41 sows, eight animals had grade 2 lesions and four animals grade 1 lesions. There was no significant difference between both groups (*p* = 0.997).

### Reproductive performance

**Fig. 5:**
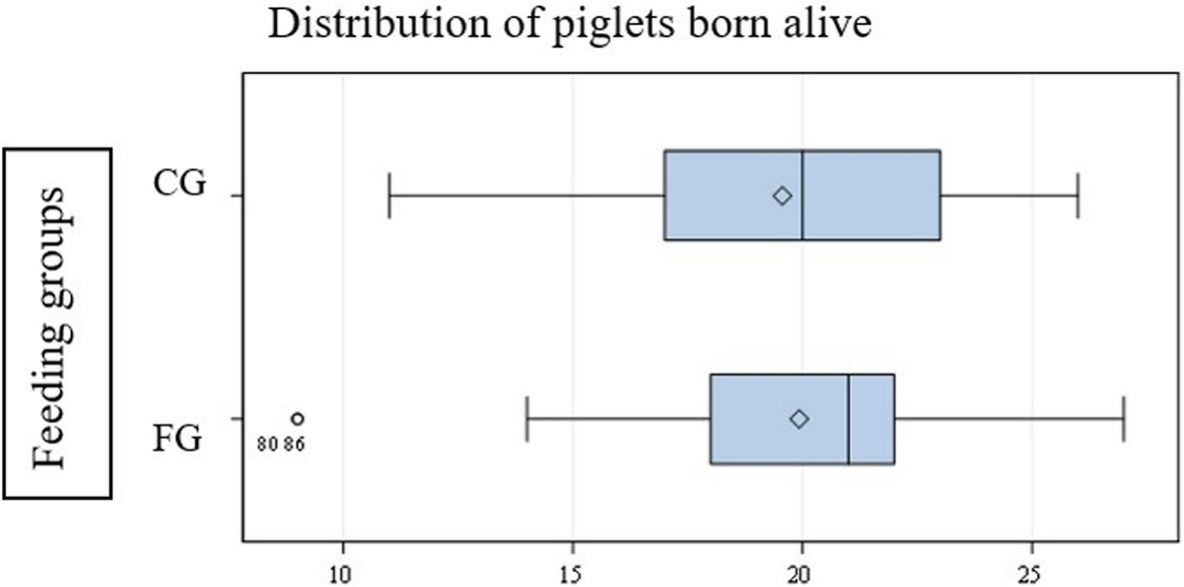
Representation of the number of piglets born alive. The upper bar shows the number of piglets born per sow in the CG. The lower bar shows the result of the FG. There was no significant difference between the two groups

The farrowing of the CG lasted almost exactly as many minutes (355.6 min) as in the FG (354.8 min)(*p* = 0.26). The number of piglets born alive (CG 19.6 (±0.6) piglets/sow vs. 19.9 (±0.6) piglets/sow in the FG) (*p* = 0.67) and the litter weight (CG 25.1 kg (±5.8 kg) vs. 25.2 kg (±4.3 kg) at the FG) (*p* = 0.76) did not show any significance (Fig. 5). The number of stillborn piglets (CG 2.0 (±2.3) vs. FG 2.6 (±2.1)) (*p* = 0.14) and their weight (CG 1.7 kg (±1.9 kg) vs. FG 2.5 kg (±2.3 kg)) (*p* = 0.10) were also similar in both groups. Again, the recording of the number of mummies showed no significant differences (CG 0.7 (±1.0) vs. FG 0.6 (±0.9)) (*p* = 0.45).

There were no significant differences between the two feeding groups. The number of weaned piglets was between 10 and 17 in both groups, which resulted in a mean number of 13.8 ± 1.6 CG vs. 13.5 ± 1.7 FG (*p* = 0.39). The litter weight in the FG (83.8 kg ± 13.1 kg) was slightly lighter than in the CG (85.6 kg ±15.3 kg) (*p* = 0.83).

Feed intake during lactation did not show any significant differences. The sows in the CG had an average daily feed intake of 5.9 kg (±0.9) vs. FG 5.7 kg (±2.0) (*p* = 0.248), with the maximum daily intake levels of 7.2 kg in the CG vs. 7.4 kg in the FG.

## Discussion

In order to prevent the sows from becoming fat during pregnancy, breeding sows are fed restrictively during this period [40]. This leads to increased feed motivation and competition during feeding [12]. The postprandial satiation is increased by the consumption of bulky fibre [17, 18]. Optimising the fibre supply was tackled by analytically characterising various fibre carriers (Analytical trial), comparing the digestibility of the test diet produced on the basis of these results (Digestibility trial), and finally recording the behaviour of sows during pregnancy as well as performance parameters for the duration of a reproduction cycle and a comparable feeding trial in practice (Field trial).

### Physical characteristics of feed components

As the usual classification of the individual feed components according to CF seems not to be sufficient, the physiochemical properties (SC, viscosity, and WBC) of various feed components were first investigated in the laboratory at the University of Applied Sciences Bingen. The findings in Table 3 clearly show that the CF values are not directly related to the WBC, viscosity and SC values. Corn germs, for example, have a CF content of 12.37%, a WBC content of 404% and a SC of 200%. In comparison, beet pulp with an analysed equal value of 12.78% CF behaved significantly differently with respect to the physical investigations. Maximum values in the WBC of 989% and SC of 675% were measured. Similarly, the values of oat fibre showed a CF content of 21.37% and for alfalfa, one of 27.76%. Alfalfa was therefore only just 6% above the CF content of oat fibre. In the context of the physical investigations, however, a much larger potential difference becomes clear. Alfalfa showed with a 338% SC, an almost twice as high SC ability compared to oat fibre with a SC of 175%. The correlation of methods was positive between WBC, WHC and SC for most ingredients and it makes sense to use the physical methods of investigation to rank fibre sources [5]. As our results and the literature show, it is necessary to use the physical parameters to qualify the fibre sources and to design a feed based on these values as well as to investigate the effects on the digestibility and the performance of the sows.

### Digestibility of a new fibre combination in diets for pregnant sows

In the present study, physical characteristics of specific fibre components were used as selection criteria for designing the diet for pregnant sows, not data on digestibility as is standard. Only fibre components with a high SC, viscosity and WBC were chosen for the sow supplementary diet.

Our study could demonstrate that replacing the CF source but using the same amount of fibre has a significant positive impact on the digestibility. A previous study using 50% alfalfa in their diet showed an increased digestibility of the fibre [29]. The disadvantage, however, of this diet was the cost efficiency, which made it impossible to use it in broad practice. A current study showed also a positive influence of high-quality fibre components on the digestibility in practice [29]. For further optimising fibre supply of pigs, physical parameters analysed by standardised, feasible methods could help to develop new dietary concepts. As a study [5] has already shown, there are different methods for investigating feed with regard to their physical parameters such as SC, viscosity and WBC. It is known that the processing of feed such as grinding has an effect on the measurement results in the laboratory [41, 42]. Furthermore, the effects of processing the feed such as pressing or heating have to be investigated. It is quite possible that individual feed components show better SC under laboratory conditions and that these are influenced or altered by processing or digestion in the animals.

### New fibre concepts in use in sows

#### Physical characteristics and behaviour

In our study, it could be proven that the use of fibres with better physical characteristics in practical rations leads to a better digestibility of the fibre but has no effect on sows’ behaviour under practical conditions. However, the animals were fed dry feed in the digestibility trial, whereas in the field trial, sows were fed with liquid feed. It cannot be excluded that possible positive effects of the designed fibre concept are cancelled out by mixing with water before feed intake. Therefore, it is possible that the effects in the feeding experiment did not differ significantly in terms of behaviour and performance.

In general, the digestibility of the fibre varies according to the botanical origin of the fibre. The highest digestibility values of DF are obtained with high pectins and/or low lignin and/or high soluble DF levels in DF (sugar beet pulp, for instance) and the lowest with high lignin and high cellulose levels in DF (straw, for instance) [6]. However, it is difficult to use the combination of DF composition as predictors of digestibility of DF in practice mainly because it ignores physical interactions between fractions or the structure of DF (Noblet and Le Goff 2001). The different WBC of the different raw fibre components influence the duration of the sows’ feed intake as well as that of gastric emptying [7, 8, 43]. In addition, a high WBC and SC provide an increased surface area for the microbes to attach to and digest the fibres [6]. Nonetheless, the age of the pigs also has an influence. Fattening pigs (digestibility trial) that are not yet fully grown do not have a fully developed colon or the microbiome necessary for the splitting of the vegetable fibre components is not yet sufficiently developed [6]. In adult sows, however, the digestibility of the fibre is much higher due to the more mature colon and its microbes [6].

Furthermore, the available energy and nutrient uptake is an essential factor modulating satiation and indicates that adding fibre to sow nutrition is only effective if the energy and nutrient supply are identical to those of a conventional diet [13]. It is advisable to see how far the fibre content in the feed can be increased so that the positive effect on the sows’ behaviour becomes more obvious.

#### Faecal consistency

Measurements of faecal consistency by penetrometer indicated no significant differences between the two groups. The higher faecal consistency values of the fibre-rich feed indicated a higher intestinal activity [28]. This could not be shown in the present study. It is remarkable that in preliminary tests, the digital penetrometer PNR12 was tested with faeces from fattening pigs. These measurements showed differences in faecal consistency after only 1 week of offering the test diet rich in components with revalued physical characteristics. Since sow faeces generally have a different consistency than those of fattening pigs, measurements with the penetrometer are more difficult and may not lead to reproducible results in every case. In order to be able to measure a correct penetration depth of the weight, the same amount of faeces must be evenly distributed in the measuring cup. However, if the material is too hard, gaps appear in the measuring cup which can only be removed by pressing down in the excrement. Nevertheless, manipulating the faeces, by pressing or homogenising, should not take place. Otherwise, the faeces will be compressed and the measurement results will be falsified.

#### Influence of fibre on body condition and activity

The BFL was measured to assess the condition of the sows in both feeding groups and the influence of the fibre. It is desirable for sows to have at least 17 mm back fat thickness at farrowing and sows are permitted to lose 3 to 4 mm of back fat during lactation [44]. Both experimental groups went into farrowing with optimal BFL values. Replacing the fibre components had no negative effects on the body condition of the sows. Low fluctuations in the live weight of the sows during the production cycle are associated with high fertility and improved longevity [19].

#### Aggression in relation to the feeding

Our results indicate once more that sows not only show a less aggressive behaviour with a fibre surplus, but also that the targeted replacement of the fibre source tends to reduce the aggressive behaviour in practice due to its better physical properties. The number of skin lesions counted weekly for the two groups were not statistically significant, but the number of category 1 and 2 lesions tended to be higher in CG.

On looking at the individual hours of video evaluation, it is noticeable that the animals showed significantly fewer interactions within the first hour to the benefit of the CG. This could be due to the fact that the feeding system could only feed one variant at a time. Therefore, the CG was always fed earlier than the FG. Since the animals were all housed in the same barn, the sound of the screw conveyor was heard when the feeding was started, to which the animals of the entire barn reacted with vocal expression and restlessness. It is quite imaginable that the significantly higher interaction in the FG was due to the sound of the feeding animals in the CG and the longer wait for the actual feeding in the FG. In contrast, in the third hour, a significant difference was shown in the interactions, as well as the number of fighting victims to the benefit of the FG. This indicates that although feeding started a short time later, the feeling of satiation within the first 2 hours produced a positive effect in the FG and earlier calmness within the group.

Sows fed a fibre-rich diet generally show reduced activity and fewer oral stereotypies after the end of the meal. They spend more time taking a daily ration through increased chewing movements and a lower intake rate compared to sows fed conventional diets. Aggressive interactions due to feed restrictions can be reduced in grouped sows [12]. The results suggest that the effects of a high-fibre diet are more effective in reducing stereotypical behaviour when the sows are younger and receive this feed over a longer period. The effect increases when gilts have received this diet over several pregnancies. One reason for this could be that stereotypical behaviour becomes more rigid and frequent over more successive pregnancies [26]. In addition to the reduction in stereotypies, the use of increased fibre content in the feed also increases the resting times of the animals [16]. In another study, it was shown that sows with limited access to straw, in combination with a high-fibre diet manipulated the soil less than those fed a lower fibre control diet [27].

Even until the end of the experiment, animals were taken out of the pens again and again since the aggression did not stop until the animals had been moved to the farrowing pen. A previous study [11] showed that liquid feeding systems, in which the pigs drink more feed than eat, reduce feed intake time by 50% compared to dry feeding systems. The animals are generally more active and show more undesirable behaviour (i.e. belly nosing in fattening pigs). Nevertheless, in another study which tested aggressions during feeding in sows between dry and liquid feeding systems, it was found that the animals in the liquid feeding system bite less during feeding [4]. Even though the liquid feeding system is considered to be more animal-friendly than the dry feeding one, there are further adjustments to be made with regard to feeding when it comes to the welfare of the animals.

Another study showed that by increasing the use of CF in the feed ration, sows kept in groups show a lower incidence of skin lesions compared to conventionally fed animals. This suggests that a fibre-rich diet in general lowers the level of aggression [12]. Especially in stables with open troughs, it is often impossible for low-ranking animals to take their feeding place undisturbed. Even after a hierarchy within a group has been established, food envy leads to new stress for the animals on a daily basis. There are numerous studies which prove that the well-being of sows can be influenced positively by the targeted use of an increased fibre amount in the feed. In group-housed sows, stereotypies can be reduced by the increase in fibre content and overall volume of feed [3].

Further studies with an optimally selected fibre fraction and an increased fibre quantity in combination should be carried out.

However, the term animal welfare should not be confused with the basic needs of an animal. Welfare is a multifactorial phenonemon. A previous study has shown that, for example, an available area 33% higher than the EU legal minimum reduced agonistic behaviour and consecutive wounds and thus induced better welfare conditions for sows living in dynamic groups (minimal legal space in the EU is 2.25 m^2^/sow) [45]. Aggressive behaviour at the time of regrouping, as well as feed envy, are normal behaviours. Positive welfare means that animals have the ability to respond appropriately (i.e. adaptively) to positive and potentially harmful (negative) stimuli [46]. The conventional management restricts the animals to such an extent that normal adaptability cannot be exercised, even though the animals are able to adapt to certain environmental conditions on a limited basis [46]. It is therefore highly important to adapt the housing conditions to the needs of the individual animals and not the other way around.

Aggression within a stable group over the entire gestational period, however, qualifies as abnormal behaviour of sows. Aggressions, like other diseases, has multifactorial causes. The well-being of the animals can be improved by the targeted use of fibres in the feed, but the improvement in animal welfare is not actively raised. It is rather an attempt to improve a parameter that can be implemented quickly. Feeding mixture can be changed ad hoc, in contrast to the type of housing, and it is important not to focus on the economic factor in feeding but on the composition with regard to animal welfare. The size of the group as well as the combination of measures needed to be taken for constructing the stable can certainly lead to improved animal welfare. Nevertheless, the feeding alone cannot lead to a situation in which sows are kept in accordance with animal welfare standards.

#### Influence of fibre on the sow’ performance

In our study, no significant differences in litter weight or number of live born piglets between the two feed groups could be observed. Also, the feed intake of the sows in lactation did not differ significantly from each other. This means that the sows` performance was not negatively affected. Previous studies have shown that sows fed with a higher fibre content in the ration consumed more water than the control animals [28]. The piglets of these sows had a higher daily weight gain in the first 5 days and were on average 0.2 kg heavier than the piglets of the CG at day 5. This is due to a better milk yield of the sows which is attributed to the additional water intake [28]. A fibre-rich diet has the positive effect that the sows can ingest larger amounts of feed during lactation. The resulting more stable milk yield in turn is responsible for the good growth rate of the piglets [12]. The increase in the number of piglets suckled by sows from the FG was also no different from that of the CG, contrary to findings in a previous study [47]. For future studies, it should be tested if a higher proportion of high-quality fibre can be used in the diet so that the positive effects become more obvious without the sows’ performance decreasing.

## Conclusions

Keeping sows in conventional sow systems offers little opportunity to the animals to follow natural eating and social behaviour during the gestation period despite group housing. Since feed intake is one of the only occupation opportunities in the stable and a rewarding resource for the animals, it is important to make use of all possibilities to improve animal welfare that are within the scope of feeding. Feed is often tested for individual properties in the laboratory and feed recommendations are then made based on those theoretical results, without considering the practicability in agriculture. Our digestibility study clearly shows that fibres with high values in SC, viscosity and WBC are more digestible than those with lower values. Many studies have shown that using higher amounts of fibre has a positive effect on the behaviour of sows. Although fewer aggressive interactions were observed in sows in the FG, we were able to show that if the fibre source is only exchanged and the amount not significantly increased, the effects do not become significantly visible. It is advisable to see how far the fibre content in the feed can be increased so that the positive effect on the behaviour of the sows becomes more obvious. However, there is often the danger that feed compositions are tested which cannot be economically implemented by farmers or which have a negative impact on the performance of the animals. The optimal approach is therefore to test the relationship between laboratory results and direct feasibility in agriculture. Furthermore, there is a need to meet all aspects of animal welfare. Feeding, livestock farming and ethology must no longer be considered separately. In this study, the first step was taken to see what can be done for animal welfare regarding animal nutrition. It must be made clear that changing the composition of feed alone does not sufficiently support animal welfare.

## Data Availability

The datasets used and/or analysed during the current study are available from the corresponding author at reasonable request.

## Abbreviations

BCS: Body condition score
BFL: Backfat layer
°C: Degree Celsius
CF: Crude fibre
CG: Control group
cm: centimetre
CO2: Carbon dioxide
DF: Dietary fibre
EIP: European innovation partnership
EU: European Union
FG: Fibre group
g: gramme
h: hour−/s
H2S: Hydrogen sulphide
kg: kilogramme
KTBL: Kuratorium für Technik und Bauwesen in der Landwirtschaft e.V
mL: millilitre
mm: millimetre
NH3: Ammonia
Ppm: Parts per million
rpm: rounds per minute
st: sensoric tested
SC: Swelling capacity
TierschutzNutztV: Tierschutz Nutztierhaltungsverordnung
VDLUFA: Verband deutscher landwirtschaftlicher Untersuchungs- und Forschungsanstalten e. V
WBC: Water binding capacity
WHC: Water holding capacity
